# A Review of Communication Practices Between a Psychiatric Inpatient Unit and an Emergency Department to Improve Patient Safety and Clinical Outcomes During Transition of Care

**DOI:** 10.1192/bjo.2025.10597

**Published:** 2025-06-20

**Authors:** Aradhana Gupta, Atinuke Olonisakin, Rutendo Maponga, Nargis Amir-Uddin, Pallavi Chandra

**Affiliations:** Black Country Mental Healthcare Foundation Trust, Sandwell, United Kingdom

## Abstract

**Aims:** Individuals with psychiatric disorders face a significantly higher risk of cardiovascular disease and other medical conditions, leading to increased morbidity and premature mortality compared with the general population. This disparity may also be partly due to diagnostic overshadowing. Effective communication between clinical settings is essential for patient safety and continuity of care whilst delays or inaccuracies in information sharing can have serious consequences.

This study aimed to evaluate the quality and timeliness of communication between an acute inpatient psychiatric unit, Hallam Street Hospital (HSH), Sandwell, Black Country Healthcare NHS Foundation Trust, and an emergency department, Midlands Metropolitan University Hospital (MMUH), West Midlands, to identify gaps and improve transitions of care.

**Methods:** A retrospective study was conducted between November 2024 and January 2025 reviewing inpatients transferred from HSH to MMUH. Patient records from the corresponding electronic systems were analysed (Rio (HSH) and Unity (MMUH)) to determine whether:

A handover document containing relevant clinical information was provided upon transfer to MMUH.

A discharge summary including a management plan was available upon patient’s discharge to HSH.

**Results:** Twelve patients were referred from HSH to MMUH during the study period with three (25%) requiring re-attendance. A limitation of this study was its small sample size due to the recent transition of the handover system.

Ten patients (83%) were accompanied by staff, while one (8%) attended alone, one (8%) accompanied by family.

Four patients (33%) were sent to MMUH with a handover document. Only one (8%) had been scanned onto Rio. None were available for viewing on Unity.

Nine patients (75%) returned to HSH with discharge summaries, however only five (42%) had been uploaded onto Rio.

The discharge summaries generally contained adequate details on the patient’s hospital course and management plan, aligned with NICE guidelines.

**Conclusion:** The audit highlighted a lack of a standardised protocol for written handover during patient transfers. While discharge summaries were electronically sent to GPs, a dedicated copy for HSH records was not consistently generated. Clinicians relied heavily on verbal handovers provided by accompanying staff or the patients themselves, increasing the risk of miscommunication and errors.

To enhance patient safety and continuity of care, we propose developing a standardised transition-of-care protocol, ensuring systematic documentation, and conducting a re-audit to assess improvements in practice.

